# Assessing Interactions between the Association of Common Genetic Variant at 1p11 (rs11249433) and Hormone Receptor Status with Breast Cancer Risk

**DOI:** 10.1371/journal.pone.0072487

**Published:** 2013-08-16

**Authors:** Qian Chen, Rongliang Shi, Weiyan Liu, Daowen Jiang

**Affiliations:** Department of General Surgery, Shanghai Minhang District Center Hospital, Shanghai Ruijin Hospital Corporation, Shanghai, People's Republic of China; The University of Texas MD Anderson Cancer Center, United States o America

## Abstract

**Background:**

The association between rs11249433 polymorphism on 1p11 and breast cancer (BC) has been widely evaluated since it was first identified through genome-wide association approach. However, the results have been inconclusive. To investigate this inconsistency, we performed a meta-analysis of all available studies dealing with the relationship between the 1p11-rs11249433 polymorphism and BC.

**Methods:**

Databases including Pubmed, SCOPUS, ISI web of knowledge, Embase and Cochrane databases were searched to find relevant studies. Odds ratios (ORs) with 95% confidence intervals (CIs) were used to assess the strength of association. The random-effects model was applied, addressing heterogeneity and publication bias.

**Results:**

A total of 15 articles involving 90,291 cases and 137,525 controls were included. In a combined analysis, the summary per-allele odds ratio (OR) for BC of 1p11-rs11249433 polymorphism was 1.09 (95% CI: 1.06–1.12; P<10^−5^). Significant associations were also observed under dominant and recessive genetic models. In the subgroup analysis by ethnicity, significantly increased risks were found in Caucasians; whereas no significant associations were found among Asians and Africans. In addition, our data indicate that 1p11-rs11249433 polymorphism is involved in BC susceptibility and confer its effect primarily in estrogen receptor-positive and progesterone receptor-positive tumors.

**Conclusions:**

In conclusion, this meta-analysis demonstrated that the G allele of 1p11-rs11249433 is a risk factor associated with increased breast cancer susceptibility, but these associations vary in different ethnic populations.

## Introduction

Breast cancer (BC), as a substantial global public health concern, is one of the most common cancers diagnosed in women and is the primary cause of death among women in both the developing and developed world [1]. Despite much investigation, the mechanism of breast carcinogenesis is still not fully understood. Although life/environment related factors, such as age at menarche, menopause, first birth age and exogenous hormone use are implicated in breast carcinogenesis [2], [3], accumulated evidence suggests that it is a complex polygenic disorder for which genetic factors play an important role in disease etiology [4], [5]. Genetic determinants including several high and moderate penetrance genes (BRCA1, BRCA2, BRIP1, CHEK2, PALB2, PTEN, and TP53) have been identified as BC susceptibility gene through the candidate gene approach in the past decade [6]. After accounting for all the known BC loci, more than 75% of the familial risk of the disease remains unexplained [7].

Recently, spectacular advance was made in identifying susceptible genes involved in breast cancer through genome-wide association strategy (GWAS) [8]–[10]. So far, genome-wide association studies (GWASs) have reported over 40 common low-penetrance variants in 25 loci that are associated with the BC risk reported in the National Human Genome Research Institute catalog [11]. More recently, a genome-wide association (GWA) study conducted in European ancestry population by Thomas et al. identified a new genetic susceptibility locus, rs11249433, at chromosome 1p11.2 was associated with BC risk [12]. Associations between the 1p11-rs11249433 polymorphism and BC have been independently replicated by subsequent studies; however, a proportion of them have produced inconsistent results. These disparate findings may be due partly to insufficient power, phenotypic heterogeneity, population stratification, small effect of the polymorphism on BC risk, and even publication biases. With the increased studies in recent years among East Asians, Africans and some other ethnic populations, there is a need to reconcile this inconsistency and to clarify the problems in previous studies. We therefore performed a meta-analysis of the published studies to clarify this inconsistency and to establish a comprehensive picture of the relationship between 1p11-rs11249433 polymorphism and BC susceptibility.

## Materials and Methods

### Literature search strategy and inclusion criteria

Epidemiological genetic association studies published before the end of Feb 2013 on breast cancer and polymorphism in the chromosome 1p11 were sought by computer-based searches from databases including Pubmed, SCOPUS, ISI web of knowledge, Embase and Cochrane databases without language restriction. Search term combinations were keywords relating to the chromosome 1p11 (e.g., “1p11”, “rs11249433”) in combination with words related to breast cancer (e.g., breast cancer’ or ‘malignant breast neoplasm’). We replaced one of those search terms each time until all possible combination mode were searched to avoid any missing literature. The titles and abstracts of potential articles were screened to determine their relevance, and any clearly irrelevant studies were excluded. The full texts of the remaining articles were read to determine whether they contained information on the topic of interest. Furthermore, reference lists of primary studies and review articles were also reviewed by a manual search to identify additional relevant publications (Checklist S1).

### Eligible studies and data extraction

Eligible studies had to meet all of the following criteria: (1) original papers containing independent data which have been published in peer-reviewed journal, (2) case–control or cohort studies, (3) genotype distribution information or odds ratio (OR) with its 95% confidence interval (CI) and *P*-value, (4) genotype distribution of control group must be consistent with Hardy–Weinberg equilibrium (HWE). The major reasons for exclusion of studies were (1) overlapping data, (2) case-only studies, (3) family-based studies and review articles.

Data extraction was performed independently by two reviewers and differences were resolved by further discussion among all authors. For each included study, the following information was extracted from each report according to a fixed protocol: first author, publication year, definition and numbers of cases and controls, frequency of genotypes, age, cigarette smoking, alcohol drinking, ethnicity, Hardy–Weinberg equilibrium (HWE) status, source of control, estrogen receptor (ER) status, progesterone receptor (PR) status, BRCA1 status, BRCA2 status and genotyping method. Studies with different ethnic groups were considered as individual studies for our analyses.

### Statistical methods

Crude ORs with 95% CIs were used to assess the strength of association between the 1p11-rs11249433 polymorphism and BC risk. The meta-analysis examined the association between the 1p11-rs11249433 polymorphism and the risk of breast cancer, for the: (i) allele contrast, (ii) recessive, and (iii) dominant models [13]. Heterogeneity across individual studies was calculated using the Q-statistic test followed by subsidiary analysis or by random-effects regression models with restricted maximum likelihood estimation [14]. Both fixed-effects (Mantel–Haenszel method) [15] and random-effects (DerSimonian–Laird method) [16] models were performed to calculate the pooled ORs. Owing to a priori assumptions about the likelihood of heterogeneity between primary studies, the random-effects model, which usually is more conservative, was reported in the text. Subgroup analyses were performed by ethnicity (Asian, Caucasian, African and others) and sample size (No. of cases ≤1000 and >1000). The Z test was used to determine the significance of the pooled OR. One-way sensitivity analyses were performed to access the stability of the meta-analysis’ results [17]. The potential publication bias was estimated using Egger’s linear regression test by visual inspection of the funnel plot [18]. If publication bias existed, the Duval and Tweedie nonparametric ‘‘trim and fill’’ method was used to adjust for it [19]. All P values are two-sided at the P  =  0.05 level. All of the statistical tests used in this meta-analysis were performed by STATA version 10.0 (Stata Corporation, College Station, TX).

## Results

### Characteristics of included studies

The combined search yielded 97 references. 82 articles were excluded because they clearly did not meet the criteria or overlapping references (Figure S1). Finally, a total of 15 eligible association studies were included involving 90,291 breast cancer cases and 137,525 controls [9], [15], [16], [21]–[41]. Of the cases, 82% were Caucasian, 12% were Asian, 5% were African descent, and 1% were of other ethnic origins. The main study characteristics were summarized in Table 1.

**Table 1:** Characteristics of studies included in a meta-analysis of the association between 1p11-rs11249433 and breast cancer.

Reference	Year	Country	Ethnicity	Cases/controls	Matching criteria	Genotyping method
He [20]	2012	Europe, USA	Caucasian	3683/34174	Ethnicity and age	TaqMan
Sueta [21]	2012	Japan	Asian	697/1394	Menopausal status and age	TaqMan
Kim [22]	2012	Korea	Asian	2257/2052	Age and region	SNP Array, TaqMan
Huo [23]	2012	Nigeria	African	1509/1383	Age	GoldenGate
Antoniou [24]	2011	Europe, Australia, USA, Canada	Caucasian	9006/8155	Ethnicity and age	TaqMan, iPLEX
Figueroa [25]	2011	Europe, Australia, USA, Canada, China	Caucasian, Asian	46036/46930	Ethnicity and age	TaqMan, iPLEX
Campa [26]	2011	USA, Europe	Caucasian, Hispanic white, Asian, African	8360/11513	Ethnicity and age	TaqMan
Jiang [27]	2011	China	Asian	1766/1853	Age and region	TaqMan
Chen [28]	2011	USA	African	3016/2745	Ethnicity and age	SNP Array
Stevens [29]	2011	Europe, Australia, USA	Caucasian	2976/4968	Ethnicity and age	iPLEX
Hutter [30]	2011	USA	African	316/7484	NA	SNP Array
Li [31]	2011	Sweden, Finland	Caucasian	1557/4584	Ethnicity, age and region	SNP Array
Bhatti [32]	2010	USA	Caucasian	774/989	Ethnicity and age	TaqMan
Long [33]	2010	China	Asian	2044/2054	Age and region	SNP Array, iPLEX
Thomas [12]	2009	USA, Poland	Caucasian	6294/7247	Ethnicity and age	SNP Array, TaqMan

NA: not applicable.

### Quantitative synthesis

**Figure 1:**
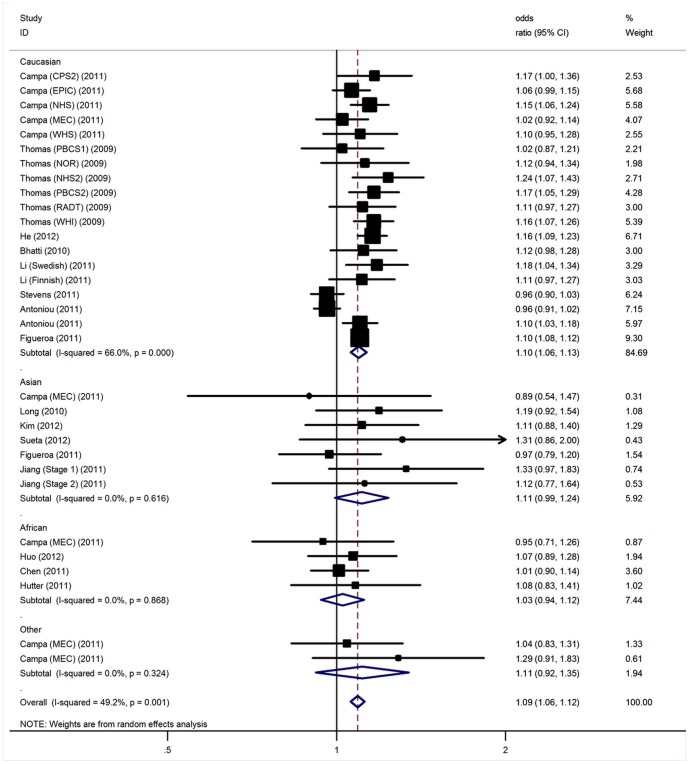
Forest plot for association of 1p11-rs11249433 polymorphism and BC risk.

Significant heterogeneity was present among the included studies of the 1p11-rs11249433 polymorphism (P<0.05). In meta-regression analysis, genotyping method (P = 0.18), sample size (P = 0.09), mean age of cases (P = 0.25) and controls (P = 0.36) did not significantly explained such heterogeneity. By contrast, ethnicity (P  =  0.002) was significantly correlated with the magnitude of the genetic effect, explaining 23% of the heterogeneity. Using random effect model, the per-allele overall OR of the G variant for breast cancer was 1.09 (95% CI: 1.06–1.12, P<10^−5^; Figure 1), with corresponding results under dominant and recessive genetic models of 1.11 (95% CI: 1.07–1.15, P<10^−5^) and 1.11 (95% CI: 1.06–1.17, P<10^−5^), respectively. When stratifying for ethnicity, significantly increased risks were found among Caucasian populations (G allele: OR  =  1.10, 95% CI: 1.06–1.13, P<10^−5^; dominant model: OR  =  1.12, 95% CI: 1.07–1.17, P<10^−5^; recessive model: OR  =  1.12, 95% CI: 1.06–1.19, P<10^−4^). However, no significant association was found for Asian and African populations with a per-allele OR of 1.11 (95% CI: 0.99–1.24, P = 0.06) and of 1.03 (95% CI: 0.94–1.12, P = 0.58), respectively. Among other ethnic populations, still no significant results were detected. Similar results were also observed for under dominant and recessive genetic models (Table 2). Subsidiary analyses of sample size yielded a per-allele OR for larger studies of 1.08 (95% CI: 1.03–1.12, P<10^−5^) and for small studies of 1.13 (95% CI: 1.08–1.18, P<10^−4^). Significant associations were also observed for both large and small studies under dominant and recessive models (Table 2).

**Table 2:** Results of meta-analysis for 1p11-rs11249433 polymorphism and BC risk.

Sub-group analysis	No. of data sets	No. of case/control	G vs. A allele				Dominant model				Recessive model			
			OR (95%CI)	P(Z)	P(Q)^a^	P(Q)^b^	OR (95%CI)	P(Z)	P(Q)^a^	P(Q)^b^	OR (95%CI)	P(Z)	P(Q)^a^	P(Q)^b^
Total	32	90291/137525	1.09 (1.06–1.12)	<10^−5^	0.001		1.11 (1.07–1.15)	<10^−5^	0.02		1.11 (1.06–1.17)	<10^−4^	<10^−4^	
Ethnicity						0.006				0.03				0.001
Caucasian	19	73771/114428	1.10 (1.06–1.13)	<10^−5^	<10^−4^		1.12 (1.07–1.17)	<10^−5^	0.001		1.12 (1.06–1.19)	<10^−4^	<10^−4^	
Asian	7	10767/10366	1.11 (0.99–1.24)	0.06	0.62		1.09 (0.97–1.19)	0.15	0.83		1.18 (0.93–1.49)	0.17	0.29	
African	4	5242/12044	1.03 (0.94–1.12)	0.58	0.87		1.02 (0.93–1.12)	0.63	0.98		1.03 (0.88–1.12)	0.72	0.31	
Other	2	511/687	1.11 (0.92–1.35)	0.28	0.32		1.11 (0.91–1.46)	0.23	0.52		1.20 (0.55–2.61)	0.64	0.19	
Sample size						0.12				0.07				0.46
<1000	17	10336/22564	1.13 (1.08–1.18)	<10^−4^	0.93		1.15 (1.09–1.21)	<10^−5^	0.98		1.15 (1.07–1.25)	<10^−4^	0.28	
≥1000	15	79955/114961	1.08 (1.03–1.12)	<10^−5^	<10^−4^		1.09 (1.04–1.15)	0.001	<10^−4^		1.10 (1.03–1.17)	0.007	<10^−4^	

a Cochran's chi-square Q statistic test used to assess the heterogeneity in subgroups.

b Cochran's chi-square Q statistic test used to assess the heterogeneity between subgroups.

### Interactions between rs11249433 and hormone receptor status with BC risk

**Figure 2:**
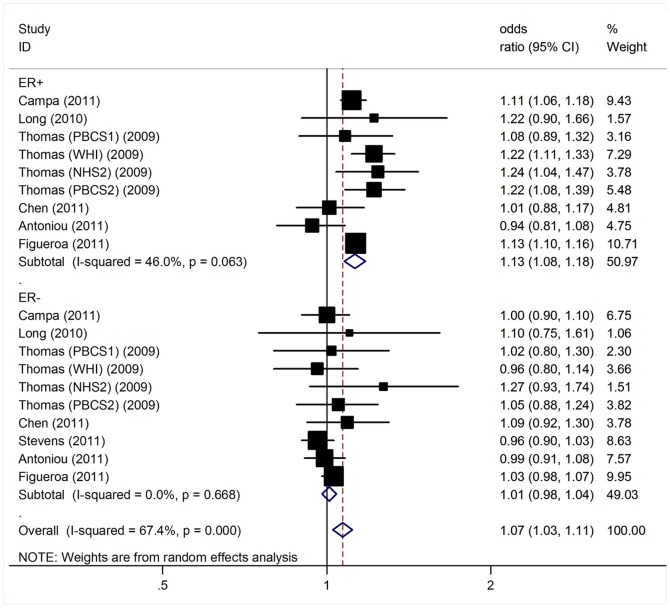
Per-allele odds ratios and 95% confidence intervals for the association between 1p11-rs11249433 and BC risk by ER status.

**Figure 3:**
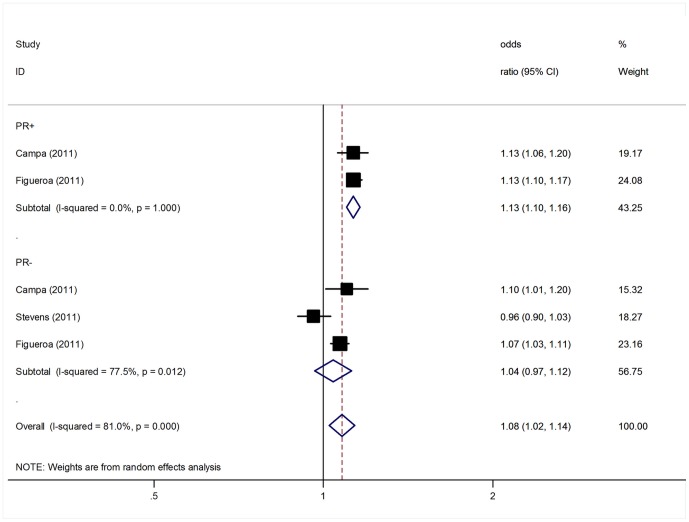
Per-allele odds ratios and 95% confidence intervals for the association between 1p11-rs11249433 and BC risk by PR status.

Since ER and PR status is one of the major markers of BC subtypes, we further performed analyses to test for differences in the associations of the polymorphism with BC risk with respect to different ER and PR status (Table 3). The minor allele of SNP 1p11-rs11249433 was associated with a significantly higher risk for ER-positive breast cancer with a per-allele OR of 1.13 (95% CI: 1.08–1.18, P<10^−5^); whereas no significant association was detected for ER-negative tumors (per-allele OR = 1.01, 95% CI: 0.98–1.04, P = 0.49; Figure 2). Similarly, a stronger association was also observed for the polymorphism with PR-positive tumors (per-allele OR = 1.13, 95% CI: 1.10–1.16, P <10^−5^) compared with PR-negative tumors (per-allele OR = 1.04, 95% CI: 0.97–1.12, P = 0.30; Figure 3).

**Table 3:** Per-allele OR for 1p11-rs11249433 variant and BC risk stratified by hormone receptor status.

Hormone receptor	Status	No. of cases/controls	OR (95%CI)	P	P(Q)^a^	P(Q)^b^
ER	Positive	25344/57465	1.13 (1.08–1.18)	<10^−5^	0.06	<10^−4^
	Caucasian only	23074/52666	1.13 (1.08–1.19)	<10^−5^	0.06	
	Negative	12170/59223	1.01 (0.98–1.04)	0.49	0.67	
	Caucasian only	10782/54424	1.01 (0.98–1.04)	0.63	0.57	
PR	Positive	11262/34668	1.13 (1.10–1.16)	<10^−5^	0.99	<10^−4^
	Negative	6577/37757	1.04 (0.97–1.12)	0.30	0.01	

### Sensitivity analyses and publication bias

A single study involved in the meta-analysis was deleted each time to reflect the influence of the individual dataset to the pooled ORs, and the corresponding pooled ORs were not qualitatively altered. Begg’s funnel plot and Egger’s test were performed to evaluate the publication bias of literatures. As shown in Figures S2, the shape of the funnel plots seemed symmetrical, suggesting no publication bias among the studies included. The statistical results still did not show publication bias (Begg test, P = 0.63; Egger test, P = 0.89, Figure S3).

## Discussion

Multiple lines of evidence support an important role for genetics in determining risk for breast cancer, and association studies are appropriate for searching susceptibility genes involved in breast cancer [34]. Nevertheless, small sample sized association studies lack statistical power and have resulted in apparently contradicting findings [35]. Meta-analysis is a means of increasing the effective sample size under investigation through the pooling of data from individual association studies, thus enhancing the statistical power of the analysis for the estimation of genetic effects [36]. In the current meta-analysis, on the basis of 15 case-control studies providing data on the 1p11-rs11249433 polymorphism and breast cancer involving 90,291 cases and 137,525 controls, we find significant association between the 1p11-rs11249433 polymorphism and breast cancer among overall and Caucasian populations. Meta-analysis is often dominated by a few large studies, which markedly reduces the evidence from smaller studies. However, in the stratified analysis according to sample size, significantly increased BC risk was found in both large and small studies.

Since ethnic group was identified as the main source of between-study heterogeneity, subgroup meta-analyses based on ethnicity was performed. Significant associations were found in Caucasians and but not for Asians and Africans, suggesting a possible role of ethnic differences in genetic backgrounds and the environment they lived in [37]. In fact, the distribution of the less common G allele varies extensively between different races, with a prevalence of ∼42% among Caucasians, ∼2% among Asians and ∼12% among African population [26]–[30]. Thus, failing to identify any significant association in Asian and African populations could be due to substantially lower statistical power caused by the relatively lower prevalence of G allele of 1p11-rs11249433. Therefore, additional studies are warranted to further validate ethnic difference in the effect of this functional polymorphism on breast cancer risk. Such result could also be due to the limited number of studies among Asian and African populations, which had insufficient statistical power to detect a slight effect or different linkage disequilibrium (LD) pattern of the polymorphism among these populations. Furthermore, study design or small sample size or some environmental factors may affect the results. It is possible that variation at this locus has modest effects on breast cancer, but environmental factors may predominate in the progress of breast cancer, and mask the effects of this variation.

Our data indicate that the association among population-based breast cancer cases is the strongest in ER-positive breast tumors. In addition, we also found that the association appeared to be much stronger for PR-positive than the PR-negative breast cancer. It is unclear whether PR status has an effect on breast carcinogenesis independent of ER status. About 65% of ER-positive breast cancers are also PR-positive, and there is a high correlation between ER and PR expression [38], [39]. Besides, the per-allele odds ratio estimates were very similar for ER+ and PR+ tumors. These findings provide further support for the notion that ER-negative and ER-positive tumors result from different etiologic pathways, rather than different stages of tumor evolution within a common carcinogenic pathway [40].

A number of factors predict breast cancer, however, detailed pathogenesis mechanisms of breast cancer remain a matter of speculation. A recent study found some evidence of increased NOTCH2 expression in breast tumors in carriers of the G allele of rs11249433 [41]. In addition, the association between rs11249433 and NOTCH2 expression was dependent on the mutational status of the tumor suppressor gene TP53 and ER status of the tumors. This suggests that either the estrogen receptor or the TP53 may have a function in the regulation of NOTCH2 expression, as the restoration of p53 expression has been shown to affect NOTCH1 expression [42], [43]. An active NOTCH pathway is important for the induction of breast stem cells to differentiate into luminal cells of breast ducts [44]. Thus, increased or persistent activation of NOTCH2 expression may favor development of ER+ breast tumors.

The strengths of this study include the very large sample size, no deviation from Hardy-Weinberg equilibrium, and the high quality of the qualified studies. However, our current study should be interpreted with several technical limitations in mind. Firstly, the vast majority of white subjects in the study are of European descent, and statistical power for analyses in other ethnicities is limited. Because the sample size was considerably smaller for African studies, the main conclusions from this manuscript are based on analyses among white European and Asian women. Future studies including larger numbers of Africans are necessary to clarify the consistency of findings across ethnic groups. Secondly, our results were based on unadjusted estimates, while a more precise analysis should be conducted if individual data were available, which would allow for the adjustment by other covariates including age, menopausal status, family history, environmental factors and lifestyle. Thirdly, the subgroup meta-analyses considering interactions between rs11249433 polymorphism and hormone receptor status were performed on the basis of a fraction of all the possible data to be pooled, so selection bias may have occurred and our results may be overinflated. Nevertheless, the total number of subjects included in this part of the analysis comprises the largest sample size so far.

In summary, findings from this meta-analysis indicate that 1p11 rs11249433 polymorphism is significantly associated with an increased risk of breast cancer, particularly in Caucasians. More work is needed to further investigate the association of the polymorphism across different ethnic populations. Besides, future studies are recommended to identify the possible gene–gene and gene–environmental interactions in this association.

## Supporting Information

Figure S1 **Flow chart of literature search for studies examining 1p11-rs11249433 polymorphism and risk of BC.**
(TIF)

Figure S2 **Begg’s funnel plot of 1p11-rs11249433 polymorphism and BC risk.**
(TIF)

Figure S3 **Test publication bias of studies of the 1p11-rs11249433 polymorphism of and BC using Egger test.**
(TIF)

Checklist S1 (DOC)
