# Supplementary figures and images for: Assessing Interactions between the Association of Common Genetic Variant at 1p11 (rs11249433) and Hormone Receptor Status with Breast Cancer Risk
Source: PLoS One. 2013 Aug 16;8(8):e72487. doi: 10.1371/journal.pone.0072487 (PMC3745461; doi:10.1371/journal.pone.0072487)

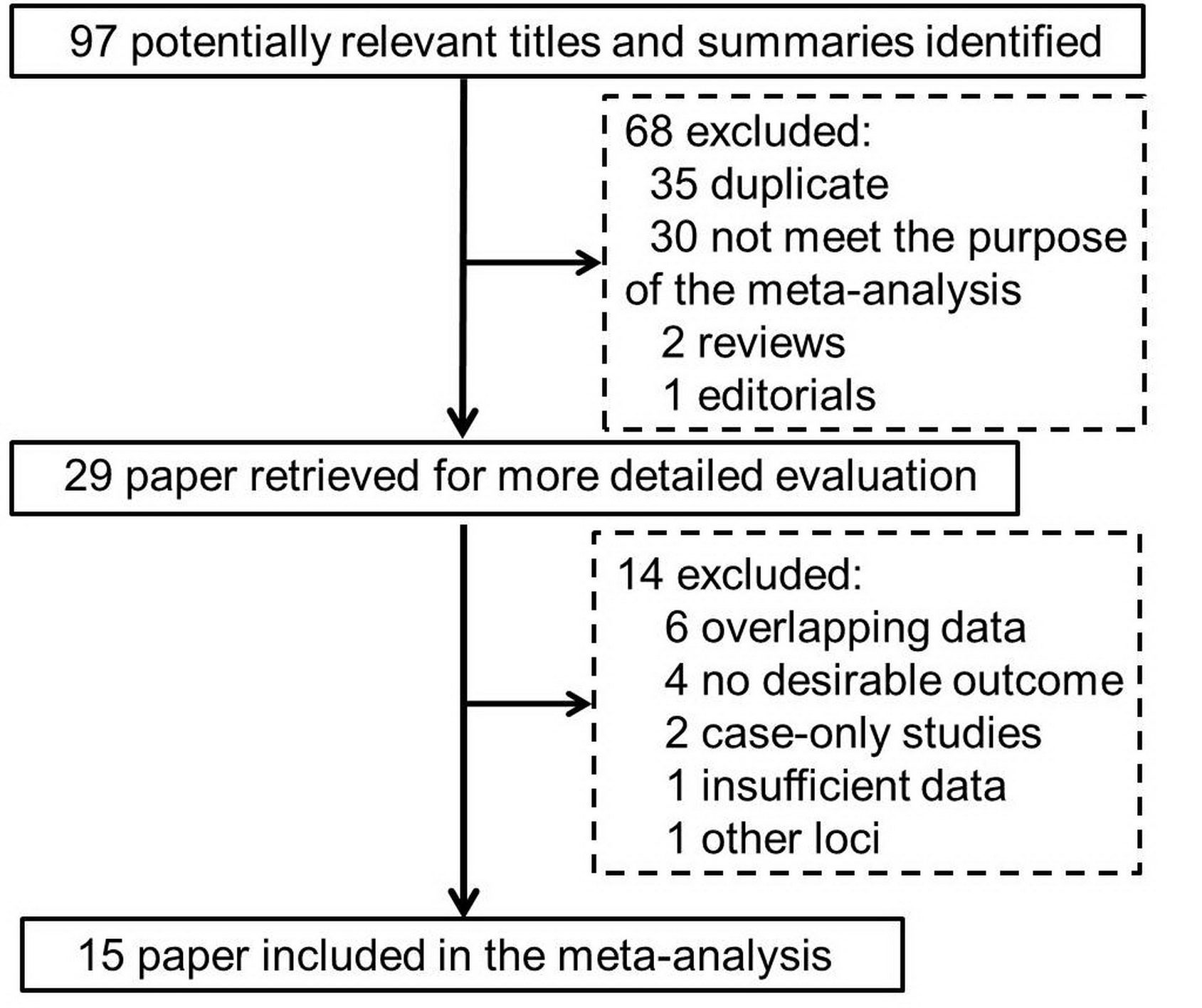

Supplement: Figure S1 — Flow chart of literature search for studies examining 1p11-rs11249433 polymorphism and risk of BC. (TIF) [file pone.0072487.s001.tif]

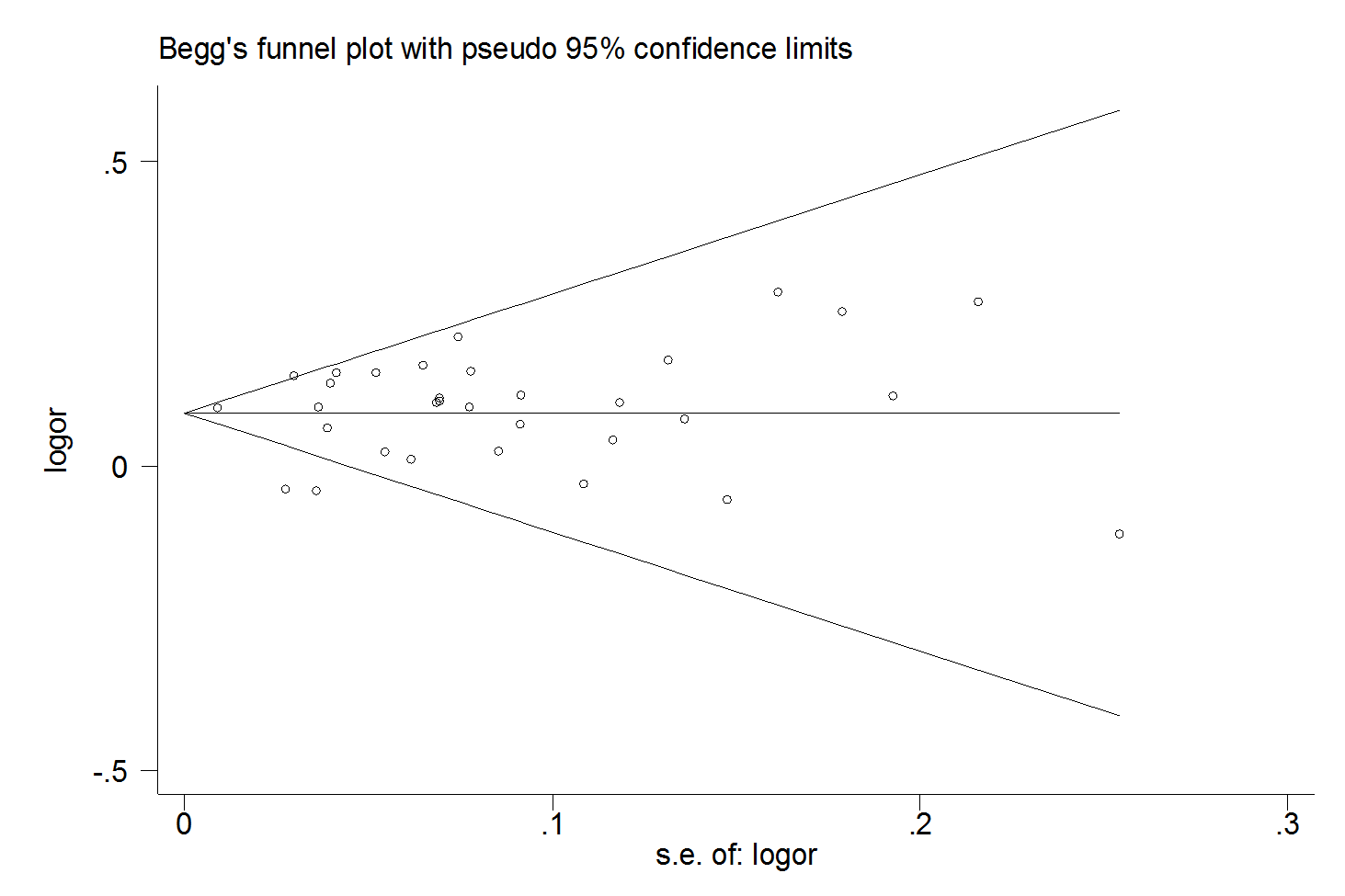

Supplement: Figure S2 — Begg’s funnel plot of 1p11-rs11249433 polymorphism and BC risk. (TIF) [file pone.0072487.s002.tif]

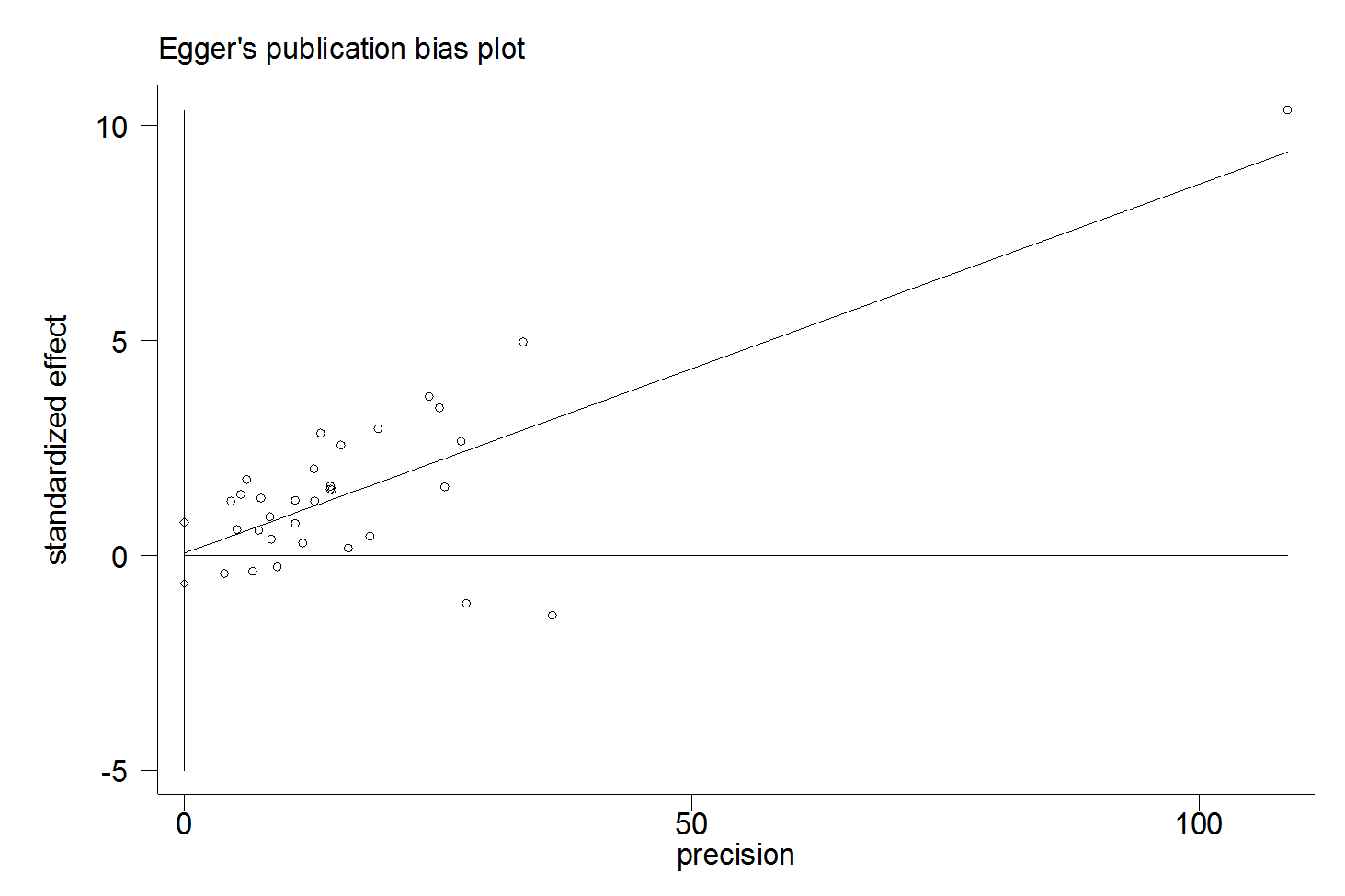

Supplement: Figure S3 — Test publication bias of studies of the 1p11-rs11249433 polymorphism of and BC using Egger test. (TIF) [file pone.0072487.s003.tif]
